# Diabetes-Specific Nutrition Algorithm: A Transcultural Program to Optimize Diabetes and Prediabetes Care

**DOI:** 10.1007/s11892-012-0253-z

**Published:** 2012-02-10

**Authors:** Jeffrey I. Mechanick, Albert E. Marchetti, Caroline Apovian, Alexander Koglin Benchimol, Peter H. Bisschop, Alexis Bolio-Galvis, Refaat A. Hegazi, David Jenkins, Enrique Mendoza, Miguel Leon Sanz, Wayne Huey-Herng Sheu, Patrizio Tatti, Man-Wo Tsang, Osama Hamdy

**Affiliations:** 1 Division of Endocrinology, Diabetes, and Bone Disease, Mount Sinai School of Medicine, New York, NY USA; 2 Department of Preventive Medicine and Community Health, University of Medicine and Dentistry of New Jersey, Newark, NJ USA; 3 Nutrition and Weight Management Center, Boston University School of Medicine, Boston, MA USA; 4 Obesity and Eating Disorders Group, State Institute of Diabetes and Endocrinology of Rio de Janeiro, Rio de Janeiro, Brazil; 5 Division of Endocrinology and Metabolism, Academic Medical Center, University of Amsterdam, Amsterdam, The Netherlands; 6 Department of General and Bariatric Surgery and Clinical Nutrition, Hospital Angeles Pedregal; Clinical Nutrition and General Surgery, Facultad Mexicana de Medicina, Universidad La Salle, México City, Mexico; 7 Research & Development, Abbott Nutrition, Columbus, OH USA; 8 Department of Nutritional Sciences, University of Toronto, Toronto, Ontario Canada; 9 University of Panama School of Medicine, Panama City, Panama; 10 Service of Endocrinology and Nutrition, University Hospital Doce de Octubre, Department of Medicine, Complutense University, Madrid, Spain; 11 Division of Endocrinology and Metabolism, Taichung Veterans General Hospital, Taichung; College of Medicine, Chung-Shan Medical University, Taichung; School of Medicine, National Yang-Ming Medical University, Taipei, Taiwan; 12 Department of Endocrinology and Diabetology, ASL RMH, Rome, Italy; 13 Division of Diabetes & Endocrinology, Department of Medicine & Geriatrics, United Christian Hospital, Hospital Authority, Hong Kong, China; 14 Division of Endocrinology, Diabetes and Metabolism, Joslin Diabetes Center, Harvard Medical School, Boston, MA USA

**Keywords:** Diabetes, Diet, Glycemic control, Nutrition, Transcultural, Prediabetes

## Abstract

Type 2 diabetes (T2D) and prediabetes have a major global impact through high disease prevalence, significant downstream pathophysiologic effects, and enormous financial liabilities. To mitigate this disease burden, interventions of proven effectiveness must be used. Evidence shows that nutrition therapy improves glycemic control and reduces the risks of diabetes and its complications. Accordingly, diabetes-specific nutrition therapy should be incorporated into comprehensive patient management programs. Evidence-based recommendations for healthy lifestyles that include healthy eating can be found in clinical practice guidelines (CPGs) from professional medical organizations. To enable broad implementation of these guidelines, recommendations must be reconstructed to account for cultural differences in lifestyle, food availability, and genetic factors. To begin, published CPGs and relevant medical literature were reviewed and evidence ratings applied according to established protocols for guidelines. From this information, an algorithm for the nutritional management of people with T2D and prediabetes was created. Subsequently, algorithm nodes were populated with transcultural attributes to guide decisions. The resultant transcultural diabetes-specific nutrition algorithm (tDNA) was simplified and optimized for global implementation and validation according to current standards for CPG development and cultural adaptation. Thus, the tDNA is a tool to facilitate the delivery of nutrition therapy to patients with T2D and prediabetes in a variety of cultures and geographic locations. It is anticipated that this novel approach can reduce the burden of diabetes, improve quality of life, and save lives. The specific Southeast Asian and Asian Indian tDNA versions can be found in companion articles in this issue of *Current Diabetes Reports*.

## Introduction

Type 2 diabetes (T2D) and prediabetes impose a huge burden of illness on developed and developing nations through high disease prevalence (6.6% overall, >10% in many countries), direct and indirect multisystem pathophysiologic effects, and financial liabilities (US$376 billion annually worldwide) [1]. This enormous disease burden can be reduced by deliberate application of interventions with proven effectiveness [2–14]. Ideally, diagnostic and therapeutic interventions should be accessible, facile, affordable, cost-effective, and culturally sensitive [1]. To improve efficiency, they can be combined in coordinated disease management programs. Lifestyle management, including physical activity and diabetes-specific nutrition therapy, is an essential and necessary component of any comprehensive care plan for diabetes [15••, 16, 17]. Care plan implementation is facilitated by clinical practice guidelines (CPGs) intended to inform clinical decisions, standardize and optimize patient care, improve outcomes, and control costs [18, 19]. Recommendations within CPGs should be evidence-based, precise, clear, relevant, authoritative, and compatible with existing norms [20, 21••]. The purpose of this report is to describe pertinent background material and the development process of a transcultural diabetes-specific nutrition algorithm (tDNA) that can facilitate portability of evidence-based recommendations to better enable their implementation and validation across a broad geographic and cultural spectrum.

## Benefits and Problems Associated with CPGs

Although CPGs may have distinct flaws or problems intrinsic to their development, interpretation, and implementation, they are useful tools to aid clinical decision making and improve patient care [21••, 22–26]. Benefits are derived from the characteristics and attributes of the CPGs. For example, authoritative guidelines are developed by expert panels from specialized areas of medicine and reflect group consensus on specific aspects of patient care. These CPGs are evidence-based, transparently incorporating relevant research findings, and contain recommendations with the greatest potential for superior clinical outcomes. Depending on the methodology used, CPGs may consider subjective factors such as risk-benefit perceptions and cost-effectiveness. They may also engage such principles as middle-range question-oriented literature searching, patient-oriented evidence, cascades of recommendations for a particular clinical question based on variations in clinical settings, multiple levels of review, and diligent screening of writers and reviewers with respect to credentialing and conflicts of interest [21••]. Through these exacting methodologies and resultant credible content, CPGs empower practitioners, patients, and the larger universe of other health care stakeholders to make better decisions regarding the applicability of care.

CPGs also have limitations [1, 19, 27]. Even if their recommendations are appropriate, their implementation and performance can be impeded by untimeliness, complexity, and/or incompatibility with other recommendations. Their adoption and adherence may be further hindered by idiosyncratic physician and patient attitudes as well as the unique characteristics of a practice setting [28]. Guidelines may not accommodate disruptions in the continuity of care that arise among health care providers, facilities, and time frames [29]. Moreover, selected recommendations may reflect only a professional perspective, which may discount patient predilections or values and compromise clinical adherence and outcomes [19, 28]. Finally, CPGs may not be able to be generalized for all patients or populations. Patient age, gender, and genomics, as well as culture, customs, and environment, must be factored into any decision to apply a particular recommendation to a particular patient in a particular setting [29]. In effect, CPGs are simply not portable across divergent clinical settings. In light of the globalization and impact of the diabetes epidemic, this significant problem must be resolved.

## Addressing the Portability Problem

Whenever possible, either de novo or the most recent up-to-date CPGs on a particular topic should be used as a resource for specific patient management issues [30]. For ease of implementation, the CPGs should be straightforward and readily understood [20]. Derivative products (i.e., decision trees, flow charts, or algorithms) can be used to reduce the complexity of comprehensive CPGs, aid comprehension, and facilitate successful implementation and validation [31–33]. Such tools not only improve the standardization of care, but also help to coordinate the activities of all members of a treatment team for patients with diabetes. A diabetes flow sheet was shown to increase CPG adherence in a recent outcomes study based on medical audits [34].

## Transcultural Factors

**Fig. 1:**
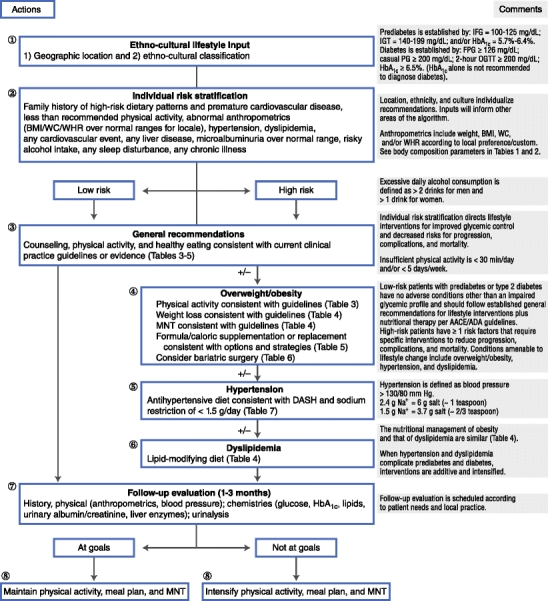
Transcultural medical nutrition algorithm for prediabetes and type 2 diabetes. AACE—American Association of Clinical Endocrinologists; ADA—American Diabetes Association; BMI—body mass index; DASH—Dietary Approaches to Stop Hypertension; FPG—fasting plasma glucose; HbA_1c_—glycosylated hemoglobin A_1c_; IFG—impaired fasting glucose; IGT—impaired glucose tolerance; MNT—medical nutrition therapy; OGTT—oral glucose tolerance test; PG—plasma glucose; WC—waist circumference; WHR—waist-to-hip ratio

To address the problem of generalizability and the effect of cultural differences among patients on a global scale, CPG development must begin with a robust decision-tree template amenable to strategic modification that does not sacrifice performance. Thus, the tDNA template was designed for the optimization of nutritional care for patients with T2D and prediabetes on a global scale (Fig. 1, Tables 1, 2, 3, 4, 5, 6 and 7) [15••, 35–40, 41••, 42]. This instrument extends evidence-based nutritional recommendations from the American Association of Clinical Endocrinologists (AACE) [15••, 43] and the American Diabetes Association (ADA) [41••] and provides nodes that can be populated with information based on geographic and ethnocultural factors for individualization and implementation at regional and local levels worldwide. The tDNA is intended to 1) increase awareness of the benefits of nutritional interventions for patients with T2D and prediabetes; 2) encourage healthy dietary patterns that accommodate regional differences in genetic factors, lifestyles, foods, and cultures; 3) enhance the implementation of existing CPGs for T2D and prediabetes management; and 4) simplify nutritional therapy for ease of application and portability.

**Table 1:** Classification of body composition by BMI, WC, and disease risk for Caucasians

	BMI, kg/m^2^	Obesity class	Disease risk	
			WC: M ≤ 40 in	WC: M > 40 in
			F ≤ 35 in	F > 35 in
Underweight	<18.5			
Normal	18.5–24.9			
Overweight	25.0–29.9		Increased	High
Obese	30.0–34.9	I	High	Very high
	35.0–39.9	II	Very high	Very high
Extremely obese	≥40	III	Extremely high	Extremely high

*BMI* body mass index; *F* female; *M* male; *WC* waist circumference

(Adapted from: Bantle JP, Wylie-Rosett J, Albright AL, et al. Nutrition recommendations and interventions for diabetes: a position statement of the American Diabetes Association. Diabetes Care. 2008;31 Suppl 1:S61–78) [41••]

**Table 2:** Classification of body composition by BMI, WC, and disease risk for Southeast Asians and Asian Americans

	BMI, kg/m^2^	Obesity class	Disease risk	
			WC: M ≤ 90 cm	WC: M > 90 cm
			F ≤ 80 cm	F > 80 cm
Underweight	<18.5			
Normal	18.5–22.9		Average	Average
Overweight	23–24.9	I	Increased	High
Obese	25.0–29.9	II	High	Very high
			Very high	Very high
Extremely obese	≥30	III	Severe	Severe

*BMI* body mass index; *F* female; *M* male; *WC* waist circumference

(Adapted from: Wildman RP, Gu D, Reynolds K, et al. Appropriate body mass index and waist circumference cutoffs for categorization of overweight and central adiposity among Chinese adults. Am J Clin Nutr. 2004;80:1129–36) [36]

(Adapted from: Appropriate body-mass index for Asian populations and its implications for policy and intervention strategies. WHO Expert Consultation. Lancet. 2004;363:157–63) [37]

**Table 3:** Physical activity guidelines for the management of diabetes

Intensity level	Physical activity
Low	Patients should be encouraged to achieve an active lifestyle and to avoid sedentary living, because physical activity and exercise provide many health benefits and facilitate glycemic control. Participation in any physical activity provides some health benefits
	For substantial benefits:
	≥150 min/week of moderate-intensity activity, or
	≥75 min/week of vigorous-intensity aerobic activity, or
	some combination of equivalent moderate/vigorous activity
Medium	Aerobic activity should be performed in episodes of ≥10 min and preferably spread throughout the week
	For additional, more extensive benefits:
	≥300 min/week of moderate-intensity activity, or
	≥150 min/week of vigorous-intensity aerobic activity, or
	some combination of equivalent moderate/vigorous activity
	additional health benefits are gained beyond this amount
High	Moderate- or high-intensity resistance exercise training for all major muscle groups, as a separate modality from aerobic exercise, has been shown to increase muscle mass and strength, alter body composition, and improve glycemic control; therefore, it should be combined with aerobic activity in each individual ≥2 days per week

Exercise should be undertaken only after cardiac clearance has been obtained

(Adapted from: US Department of Health and Human Services. 2008 Physical activity guidelines for Americans. 2008. http://www.health.gov/paguidelines/guidelines/summary.aspx. Accessed June 22, 2011) [35]

**Table 4:** AACE/ADA nutritional guidelines for the management of diabetes

Hypocaloric (weight loss) diet: 250–1000 kcal/d deficit
Target: decrease weight by 5% to 10% for overweight/obese, 15% for class 3 obesity
Target: decrease BMI by 2 to 3 units
Carbohydrates (preferably low-glycemic index): 45% to 65% daily energy intake and not less than 130 g/d in patients on low calorie diet
Protein: 15% to 20% daily energy intake
Dietary fat: <30% daily energy intake
Saturated fat: <7% daily energy intake
Cholesterol: <200 mg/d
Fiber: 25–50 g/d
Trans fats: minimize or eliminate

*AACE* American Association of Clinical Endocrinologists; *ADA* American Diabetes Association; *BMI* body mass index

(Adapted from: National Guideline Clearinghouse. Guideline synthesis: Nutritional management of diabetes mellitus. 2009. http://www.guideline.gov.syntheses/synthesis.aspx?id+16430. Accessed June 22, 2011.) [40]

**Table 5:** Diabetes-specific (glycemia targeted specialized) nutrition formulas for the management of prediabetes and diabetes

Overweight/obese	Use 2 to 3 diabetes-specific nutrition formulas^a^ as part of a reduced calorie meal plan, as a calorie replacement for meal, partial meal, or snack (grade C; LOE 3)	
	Calorie goals:	
	<250 lb = 1200 to 1500 calories	
	>250 lb = 1500 to 1800 calories	
	Calories from diabetes-specific nutrition formulas	
	Calories from other healthy dietary source	
Normal weight	Uncontrolled diabetes	1 to 2 diabetes-specific nutrition formulas per day to be incorporated into a meal plan, as a calorie replacement for meal, partial meal, or snack (grade D; LOE 4)
	HbA_1c_ > 7%	
	Controlled diabetes	Use of diabetes-specific nutrition formulas should be based on clinical judgment and individual assessment^b^ (grade D; LOE 4)
	HbA_1c_ ≤ 7%	
Underweight	Use diabetes-specific nutrition supplements^c^ 1 to 3 units/d per clinical judgment based on desired rate of weight gain and clinical tolerance (grade D; LOE 4)	

LOE 1: data defined as conclusive results from prospective, randomized controlled trials that have large subject populations representative of the target population and results that are easily generalized to the target population. Data also include results from meta-analyses of randomized controlled trials, results from multicenter trials, and “all or none” evidence; LOE 2: data include conclusive results from individual randomized controlled trials that have limited subject numbers or target population representation; LOE 3: data include all other conclusive clinical findings from nonrandomized studies, studies without controls, and nonexperimental or observational studies. These data may require interpretation and, by themselves, are not compelling; LOE 4: data are defined as information based solely on experience or expert opinion and are not necessarily substantiated by any conclusive scientific data. Frequently, only LOE 4 data are available

^a^Diabetes-specific nutrition formulas are nutritional products used as replacement for meals, partial meals, or snacks to replace calories in the diet

^b^Individuals who may have muscle mass and/or function loss and/or micronutrient deficiency may benefit from diabetes-specific nutrition supplements. Individuals who need support with weight maintenance and/or a healthy meal plan could benefit from diabetes-specific nutrition

^c^Diabetes-specific nutrition supplements are complete and balanced nutritional products used in addition to a typical meal plan, to help promote increased nutritional intake

*HbA*_*1c*_ glycosylated hemoglobin A_1c_; *LOE* level of evidence

**Table 6:** Criteria for bariatric surgery for the management of diabetes

BMI ≥ 40 kg/m^2^ (about 100 lb overweight for men and 80 lb for women) or
BMI 35–39.9 kg/m^2^ and an obesity-related comorbidity, such as T2D, coronary heart disease, or severe sleep apnea
BMI 30–34.9 kg/m^2^ under special circumstances
According to the International Diabetes Federation, bariatric surgery should be considered an alternative treatment option in patients with a BMI of 30–35 kg/m^2^ when diabetes is not adequately controlled by a medical regimen and especially when there are cardiovascular disease risk factors
Consideration may be given to laparoscopic-assisted gastric banding in patients with T2D who have a BMI > 30 kg/m^2^ or Roux-en-Y gastric bypass for patients with a BMI > 35 kg/m^2^ to achieve at least short-term weight reduction
And for each of the above:
Failure to achieve and sustain weight loss after attempts at lifestyle modification
Tolerable operative risks
Understanding of operation
Commitment to treatment and long-term follow-up
Acceptance of required lifestyle changes

*BMI* body mass index; *T2D* type 2 diabetes

(Adapted from: Weight-control Information Network—an information service of the National Institute of Diabetes and Digestive and Kidney Diseases (NIDDK). Bariatric surgery for severe obesity. 2009. http://win.niddk.nih.gov/publications/gastric.htm. Accessed November 14, 2011) [38]

(Adapted from: International Diabetes Federation. Bariatric surgical procedures and interventions in the treatment of obese patients with type 2 diabetes: a position statement from the International Diabetes Federation Taskforce on Epidemiology and Prevention. http://www.idf.org/webdata/docs/IDF-Position-Statement-Bariatric-Surgery.pdf. Accessed June 27, 2011) [39]

(Adapted from: Handelsman Y, Mechanick JI, Blonde L, et al. American Association of Clinical Endocrinologists Medical Guidelines for Clinical Practice for developing a diabetes mellitus comprehensive care plan. Endocr Pract. 2011;17 Suppl 2:1–53) [15••]

**Table 7:** Antihypertensive diet: daily nutrient goals used in the DASH studies

Carbohydrate	55% of calories
Total fat	27% of calories
Protein	18% of calories
Saturated fat	6% of calories
Cholesterol	150 mg
Fiber	30 g
Sodium	1500 mg
Potassium	4700 mg
Calcium	1250 mg
Magnesium	500 mg

*DASH* Dietary Approaches to Stop Hypertension

Based on a 2100-calorie eating plan

(Adapted from: US Department of Health and Human Services. Your guide to lowering your blood pressure with DASH. NIH publication no. 06-408. 2006. http://www.nhlbi.nih.gov/health/public/heart/hbp/dash/new_dash.pdf. Accessed June 22, 2011) [42]

## Methodology to Develop the tDNA

The methods and procedures used to develop the tDNA are widely recognized as state-of-the-art within medical organizations and were rigorously applied throughout this endeavor. The task force chair initiated the project via live meetings and telephone or digital communications. Internationally respected health care experts in diabetes and nutrition from Brazil, Canada, China, Mexico, The Netherlands, Panama, Spain, Taiwan, and the United States were identified through literature searches and peer recommendations. Each expert was contacted, briefed on the project, and questioned about his or her current activities and interest in participating in the program. Based on responses, invitations were extended until a complement of specialists, sufficient for advisory activities, accepted the request to be included in the task force.

Members of the task force provided data, culturally meaningful information, and expert opinion to guide algorithm development. During a meeting in New York City on November 12 to 13, 2010, members discussed clinical evidence and the influence of various diabetes risk factors and comorbidities (cardiovascular events, obesity, hypertension, and dyslipidemia) in the construction of the tDNA template. Task force members also deliberated over the relative merits of specific metrics (body weight, waist-to-hip ratio [WHR], fasting blood glucose, and glycosylated hemoglobin [HbA_1c_]) and nutritional therapies (foods, diets, and calorie supplementation and replacement with prepared diabetes-specific formulas) that would be cited in the template. Diabetes-specific formulas (glycemia-targeted specialized nutrition formulas) may be used for calorie replacement or supplementation as part of medical nutrition therapy (MNT). Transcultural factors influencing dietary practices, food choices, and diabetes health care interventions were also considered. For example, energy-dense fast foods are ubiquitous but may take different forms throughout the world. Likewise, healthy foods take different forms based on geography and seasonality. Table 8 lists common international foods and their glycemic indices [44]. Such information becomes essential at the local level to make nutritional therapy meaningful.

**Table 8:** Common international foods and glycemic indices

Carbohydrate foods	Glycemic index		Glycemic index
Common foods		Fruits	
White wheat bread	75	Apple	36
Whole wheat bread	74	Banana	51
Multigrain bread	53	Dates	42
Wheat roti	62	Mango	51
Chapati	52	Orange	43
Corn tortilla	46	Peaches	43
White rice	73	Pineapple	59
Brown rice	68	Watermelon	76
Barley	28	Vegetables	
Corn	52	Potato, boiled	78
Spaghetti	49	Potato, instant mash	87
Rice noodles	53	Potato, fried	63
Udon noodles	55	Sweet potato	63
Couscous	65	Carrots, boiled	39
Dairy products		Pumpkin, boiled	64
Whole milk	39	Plantain	55
Skim milk	37	Taro, boiled	53
Soy milk	37	Vegetable soup	48
Rice milk	86	Legumes	
Ice cream	51	Chickpeas	28
Yogurt	41	Kidney beans	24
Cereals		Lentils	32
Cornflakes	81	Soy beans	16
Rolled oat meal	55	Snacks	
Instant oat meal	79	Chocolate	40
Rice congee	78	Popcorn	65
Muesli	57	Potato chips	56
Millet porridge	67	Soda	59
Biscuits	69	Rice crackers	87

Glycemic index (GI) ranks carbohydrates according to their effect on blood glucose levels. High GI = ≥70; medium GI = 56–69; low GI = ≤55

(Adapted from: Atkinson FS, Foster-Powell K, Brand-Miller JC. International tables of glycemic index and glycemic load values: 2008. Diabetes Care. 2008;31:2281–3. 45. Baker R, Feder G. Clinical guidelines: where next? Int J Qual Health Care. 1997;9:399–404) [44]

The evidence supporting task force recommendations was rated and assigned a numerical descriptor according to levels of scientific substantiation provided by the 2010 AACE protocol for the development of CPGs (Table 9) [21••]. The cumulative information was then codified using an alphabetic descriptor (grade A, B, C, D), reflecting the respective strength of the recommendation [21••]. The data and information used to construct the algorithm, as well as the included recommendations, closely reflect similar information and grading found in the diabetes nutrition sections of the AACE [15••, 43] and ADA [41••] CPGs.

**Table 9:** Levels of substantiation and their respective numerical and semantic descriptors

Level of evidence	Study design or information type
1	RCTs
1	Meta-analyses of RCTs
2	Nonrandomized RCTs
2	Meta-analyses of nonrandomized RCTs
2	Prospective cohort studies
2	Retrospective case–control studies
3	Cross-sectional study
3	Surveillance study
3	Consecutive case series
3	Single case report
4	Expert consensus
4	Expert opinion based on experience
4	Theory-driven conclusions
4	Experience-based information
4	Review

*RCT* randomized controlled trial

(Adapted from: Mechanick JI, Camacho PM, Cobin RH, et al. American Association of Clinical Endocrinologists Protocol for Standardized Production of Clinical Practice Guidelines—2010 update. Endocr Pract. 2010;16:270–83) [21••]

Following the initial task force meeting, a subcommittee reviewed a meeting transcript to adopt points of agreement and resolve points of disagreement to achieve consensus on all major topics of discussion. Subsequently, all task force members received abstract summaries of the proceedings for their review and subsequent modification or approval. Consensus recommendations were discussed at a second task force meeting held on June 17 to 18, 2011 in New York City. At that time, recommendations were critiqued, refined, and prepared for transcultural adaptation by an expanded task force team that included additional experts from Canada, India, and Spain.

## Transculturalization Standards

The transculturalization of CPGs addresses problems that arise when recommendations are considered for implementation in an environment beyond that of the sponsoring individuals or organization [45]. In regard to nutrition therapy, transcultural factors relate to genetic differences within a given population, food preferences, religious practices, socioeconomic status, and others. Attributes of the transculturalization process should include evidence-based methodology, scientific rigor, transparency, relevance, and authority commensurate with the original CPGs [30, 46–50]. To obtain the cooperation and acceptance of key regional stakeholders in the implementation of the CPGs, their participation in the transculturalization process is essential. Likewise, a mechanism to have regional experts train local stakeholders and then continue the iterative process is vital, along with a validation and evaluation process to further modify the CPGs, if needed [50, 51]. To accommodate patient opinion and choice, subjective patient preferences for health care interventions that are locally available should be considered [52–54], as well as cascades of alternative strategies for a specific action [55, 56] and patient aids to inform their decisions [53].

## Transculturalization of the Diabetes-Specific Nutrition Algorithm

On July 8, 2011, clinical experts from the Philippines, Hong Kong, Indonesia, Malaysia, Taiwan, Singapore, and Thailand met in Taipei, Taiwan to learn about the tDNA and begin the process of adapting the algorithm to their territories. This transcultural group was composed of endocrinologists, dietitians, and primary care practitioners who represented the health care specialties serving the patient populations that were targeted for the adaptive process. During the meeting, participants received information on diabetes and lifestyle modification, nutrition therapy and related clinical outcomes, and tDNA program goals and objectives. A point-by-point review of the algorithm was undertaken to explore pathways, content, and supportive evidence and to elicit information for the cultural adaptation of the algorithm and related recommendations for Southeast Asian patients. Subcommittee meetings were subsequently held in Taiwan (July 18, 2011) and India (September 23, 2011) to explicitly populate the nodes of the algorithm and the cells of the calorie supplement/replacement matrix (Table 5) with specific transcultural information and recommendations for their respective regions. Companion articles in this issue of *Current Diabetes Reports* describe the adaptive process and related output [57, 58].

## Results

An amalgam of the deliberations and conclusions of the expanded international task force is presented here, displaying the consensus composite template of the tDNA, which is being used in the transculturalization process. Adaptations will be considered in ongoing regional meetings until provincial versions of the algorithm are available for local implementation and validation throughout the world.

### R1

Diets, meals, and foods influence glycemic status and the risk of diabetic complications (grade A) [16, 59–61].

### R2

MNT is important and should be implemented as an essential component of comprehensive management programs for all patients with T2D and prediabetes (grade A) [41••, 43, 62–67].

### R3

Diets should be based on individual risk factors for impaired glucose tolerance, obesity, hypertension, and dyslipidemia (grade A) [64, 65, 67, 68].

### R4

Cultural factors should guide the selection of local foods and meals to adhere with general nutrition recommendations from the AACE and ADA (grade D) [1, 41••, 43].

### R5

Diabetes-specific formulas may be used for calorie replacement or supplementation as part of MNT (grade A) [41••, 43, 69•, 70, 71]. Prepared and packaged diabetes-specific formulas contain nutrients that are designed to facilitate glycemic control [69•]. Such nutrients include modified maltodextrin, fructose, fiber, soy protein, monounsaturated fatty acids, and antioxidants. Clinical studies have demonstrated improvements in glycemic profiles and reductions in disease complications among patients who consume prepared formula products as part of MNT. For patients with low body mass index (BMI) and/or insufficiency states, such as the elderly, caloric supplementation is helpful for weight gain, amelioration of nutritional deficiencies, and prevention of diabetic complications [69•, 72–75]. For patients with normal or elevated BMI, caloric replacement is helpful to achieve weight loss, greater metabolic control, and avoidance of subtle deficits of vitamins or other nutrients that can accompany simple calorie restriction [69•]. Intensification or reduction in the number of replacements and supplements is a stepped process based on clinical appraisals and modification of regimens to meet individual patient goals [73].

### R6

Geographic location and ethnocultural classifications should be used to tailor the algorithm for specific patient populations. Risk factors are the leading determinants of patient pathways and related recommendations; one or more risk factors identify patients who are more likely to experience disease progression and/or complications (grade A) [75–77]. Dietary modification can mitigate the following risk factors: T2D or prediabetes, excessive weight or obesity, hypertension, and dyslipidemia (grade A) [64, 65, 67].

### R7

Anthropometric measures—BMI, waist circumference (WC), or WHR—should be used to assess body composition and risk of progression [41••]. Although each of these measures has merit in clinical practice, differences in values and interpretations arising from phenotypic and cultural differences among populations have confounded global standardization. Likewise, difficulty using some methods (eg, WHR) has created regional preferences in medical practice and influenced general recommendations. Consequently, BMI and WC were chosen as the preferred prioritized measures of body composition in the algorithm. Values for normal and abnormal composition can be adjusted via ethnocultural inputs for local applicability (grade B) [78, 79].

### R8

Irrespective of patient risk factors, lifestyle intervention mandates professional counseling, physical activity, and healthy eating patterns consistent with current CPGs or evidence (grade A) [41••, 43]. Professional counseling may be impeded by local attitudes and costs as well as the lack of perceived value by patients who may be economically disadvantaged. Although both low-risk and high-risk patients should comply with these basic recommendations, high-risk patients should intensify their efforts according to their specific needs and conditions.

### R9

A registered dietitian (RD) who is familiar with the components of MNT should be involved in patient management (grade B) [41••]. Long-term changes in behavior are difficult to achieve for many patients. To assist implementation, physicians should encourage and support patients through the behavior modification process. However, physicians are limited by time and experience in behavior modification techniques. Therefore, the use of other health care professionals with expertise in patient self-management may be necessary. Research has shown that lifestyle case management by RDs can improve health outcomes among patients with T2D [80, 81•, 82]. In cultures and regions (e.g., Hong Kong) where patients may oppose or decline nutrition consultation by health care providers who are not physicians, we encourage physicians to develop skills in nutrition medicine by participating in appropriate continuing medical education.

### R10

Patients should be encouraged to lead an active lifestyle and avoid sedentary living, as physical activity and exercise independently confer health benefits and facilitate glycemic control (grade A) [35, 83–85]. Substantial benefit is achieved with ≥150 min per week of moderate activity or ≥75 min per week of vigorous aerobic activity [83–86]. Resistance exercise training, as a separate modality from aerobic exercise, can increase muscle mass and improve glycemic control and should be combined with aerobic activity (grade D) [87]. Additional time spent on any physical activity can augment benefit and represents an intensification strategy for patients with higher risk stratification or those who do not achieve their goals with less intense activity.

### R11

MNT was introduced in 1994 by the American Dietetic Association to better express the concept of therapeutic nutrition [65, 88]. It consists of specific nutritional interventions that include assessment, counseling, and dietary modification, with and without specialized nutritional formulas for calorie supplementation or replacement [65]. Diabetes-specific MNT has been considered a cornerstone of diabetes treatment because it can improve glycemic profiles and reduce the risk of disease complications [65, 67, 89]. Formalized recommendations for T2D in the medical literature include the following: carbohydrates, preferentially from low-glycemic index foods, for 45% to 65% of daily energy intake and not less than 130 g/day in patients on low-calorie diets (grade D) [41••, 43, 90, 91]; fats for less than 30% of daily energy intake (grade D) [41••, 43, 90]; saturated fat for less than 7% of daily energy intake (grade A) [41••, 43, 90, 92]; protein for 15% to 20% of energy intake and not less than 1 g/kg in patients with normal kidney function (grade D) [41••, 43]; cholesterol restricted to less than 200 mg daily (grade A) [41, 43, 90, 92]; *trans* fats eliminated or reduced to minimal intake (grade D) [41••, 43, 90, 92]; and fiber for 25 to 50 g daily (grade A) [41••, 43, 90].

### R12

Overweight or obese patients should adhere to these guidelines and try to achieve a gradual weight loss of 5% to 10% by reducing caloric intake, for a total daily deficit of 250 to 1000 kcal (grade A) [15••, 16, 17, 41••, 43, 70, 93, 94]. Patients with class 3 obesity should shed 15% of body weight (grade D) [15••, 16, 17, 41••, 43, 70, 93, 94]. BMI should be decreased by 2 to 3 units (grade D) [41••, 43, 70, 93, 94]. Any amount of weight loss is associated with metabolic benefits, even if clinical targets are not met.

### R13

Bariatric surgery may be considered for patients with T2D and obesity who 1) fail to respond to lifestyle and pharmacologic interventions; 2) meet established criteria related to body composition, comorbidities, and surgical risk; and 3) commit to durable lifestyle changes and follow-up evaluations [95–98]. The recent statement by the International Diabetes Federation on criteria for bariatric surgery should be considered when making treatment decisions [39].

### R14

Patients with T2D or prediabetes complicated by hypertension require further nutritional management. Sodium intake should already be limited to 1,500 mg/day per recent recommendations provided by the Dietary Guidelines for Americans, 2010 (grade A) [99–102, 103•, 104]. The principles of the Dietary Approaches to Stop Hypertension (DASH) diet, particularly increasing the consumption of fresh fruits and vegetables, should also be incorporated into the patient’s diet.

### R15

Patients with lipid abnormalities must pay closer attention to fat intake based on their dyslipidemic profiles and may benefit from viscous fibers and plant sterols (grade A) [91, 92, 105–111]. The reduction of simple sugars and alcohol is important for patients with hypertriglyceridemia.

### R16

When multiple comorbidities exist in a patient with T2D or prediabetes, recommended interventions are applied simultaneously and at a higher level of intensity, but individual patient factors, such as potential for adherence, adverse effects, and dietary customs and practices, are also taken into account (grade D).

### R17

Follow-up evaluations for all patients should occur at appropriate intervals depending on need (grade D). Assessments should include a history and physical examination (anthropometrics, blood pressure); blood chemistries (glucose, HbA_1c_, lipids, renal function, and liver enzymes depending on clinical status); and urinary microalbumin determination. Improvements in disease states based on follow-up assessments create an opportunity to diminish the intensity of interventions and to spare resources. Although deterioration of clinical status creates a need to increase the intensity of interventions, it also creates an opportunity to search for ways to improve care and possibly adherence.

## Discussion

Within North America, the national prevalence of diabetes and prediabetes ranges from 10.1% in Mexico to 11.6% in Canada and 12.3% in the United States [1]. The global prevalence is significantly lower, at 6.6%, but rates in the Arab countries are much higher at 13.4% (Oman) to 18.7% (United Arab Emirates) [1]. These figures reflect accurate estimates of the size of affected populations, but do not convey the complexities that underlie the epidemiology of the disease. Hidden behind these numbers are the details that illuminate the nature of the problem. Today, societies are heterogeneous not only in North America, but in many other places around the world. Continents, countries, states, municipalities, and even neighborhoods can be characterized by ethnicity, customs, mores, habits, and beliefs. All of these demographics influence personal choices that contribute to health or illness and the prevention or development and perpetuation of diabetes.

Health care providers must understand the culture of diabetes to effectively manage their patients with diabetes. Although general cultural sensitivity training is offered in medical school and through postgraduate activities, most practitioners are woefully deficient in the knowledge and skill to maneuver medically through the intricacies of a diverse patient population [112–115]. Clinical guidance rarely cites the cultural differences that truly matter in the delivery of health care. This may be especially true—and particularly important—with respect to diabetes, a disease that is intimately associated with lifestyles and foods. For these reasons, the international task force sought to identify and incorporate regional differences in genetic factors, diet, exercise, and culture into CPGs for nutrition therapy in T2D and prediabetes.

For example, based on clinical evidence, it was abundantly clear to the task force members that a principal cause of obesity and diabetes was the consumption of energy-rich or fast foods. Such foods are characterized by high concentrations of fats and refined carbohydrates with high glycemic indices. Describing foods in terms of composition, however, may limit comprehension and corrective action. Instead, citing examples that are culturally meaningful (e.g., fried white rice with pork drippings or quarter-pound cheeseburgers with french fries and soda) may be more relevant.

Assessing an individual for diabetes risk seems relatively straightforward until one realizes that Southeast Asians tend to develop the disease at a younger age and lower BMI. Disease management in Southeast Asian patients is also complicated by a greater prominence of postprandial hyperglycemia and renal complications.

Economics and education affect the likelihood that a health care intervention, regardless of its benefit, will be adopted and faithfully used by a particular segment of society. Task force members noted that the acceptance of dietitians or nutrition counselors, for instance, is low in Southeast Asia because the importance of such individuals is probably undervalued or misunderstood, not only by patients but occasionally by physicians, too. The cost and relative inaccessibility of these allied professionals may impede their participation in health care, especially when community resources are not available for targeted education. Moreover, broader education in the form of CPGs for comprehensive care of patients with diabetes as well as specific lifestyle interventions are needed worldwide, but guidance comes mostly from developed countries, especially Europe and North America, where interpretation of sophisticated recommendations is easier than in developing non-English-speaking nations. Couple this problem to a lack of familiarity with nutrition therapy in general and prepared liquid formulas in particular, and the likelihood of effective nutrition management is greatly reduced.

In response to these problems, we developed the tDNA. It has four major strengths: 1) simplicity that fosters not only an understanding of diabetes-specific nutrition therapy but also the cultural adaptation necessary for worldwide implementation; 2) incorporation of advice from national and international associations and respected publications; 3) inclusion of important clinical evidence and experience from multinational health care stakeholders who contributed to the developmental process; 4) openness to scientific and cultural adaptation.

Dietary and culinary habits within many diverse global communities must still be identified, and guidance must be further tailored along ethnic and cultural lines. To reach this end point within the tDNA program, diabetes and nutrition experts from around the world continue to be organized, familiarized with program goals, and invited to contribute to the transculturalization process. Adapted versions of the algorithm have already emerged for Southeast Asian and Asian Indian populations [57, 58]. Implementation will require education initiatives and follow-up assessments to determine if clinical benefit is truly achieved. These activities are currently being planned within the tDNA program. If found to be successful in optimizing comprehensive diabetes care, the tDNA and related educational resources will be made available for widespread dissemination and worldwide implementation.

## Conclusions

The algorithm presented here incorporates established standards used for CPG development, adaptation, and implementation. It is comprehensive and authoritative, yet brief and easy to use. Importantly, it is designed for simplicity and global cultural adaptation, or transculturalization. In large part, it conveys established clinical guidance from highly respected organizations for nutrition therapy and lifestyle management. It also references calorie augmentation or replacement with diabetes-specific liquid meals, a relatively novel addition to traditional nutrition therapy. It remains open to modification of content and context.
